# The Relationship between Infectious Diseases and Housing Maintenance in Indigenous Australian Households

**DOI:** 10.3390/ijerph15122827

**Published:** 2018-12-11

**Authors:** Shahmir H. Ali, Tim Foster, Nina Lansbury Hall

**Affiliations:** 1 Krieger School of Arts and Sciences, Johns Hopkins University, Baltimore, MD 21218, USA; sali43@jhu.edu; 2 Institute for Sustainable Futures, University of Technology Sydney, Sydney 2007, Australia; tim.foster@uts.edu.au; 3 School of Public Health, The University of Queensland, Brisbane 4006, Australia

**Keywords:** housing, health, Australia, aboriginal peoples, infectious diseases, maintenance and repair, crowding, policy, remote communities

## Abstract

This research aimed to identify systemic housing-level contributions to infectious disease transmission for Indigenous Australians, in response to the Government program to ‘close the gap’ of health and other inequalities. A narrative literature review was performed in accordance to PRISMA guidelines. The findings revealed a lack of housing maintenance was associated with gastrointestinal infections, and skin-related diseases were associated with crowding. Diarrhoea was associated with the state of food preparation and storage areas, and viral conditions such as influenza were associated with crowding. Gastrointestinal, skin, ear, eye, and respiratory illnesses are related in various ways to health hardware functionality, removal and treatment of sewage, crowding, presence of pests and vermin, and the growth of mould and mildew. The research concluded that infectious disease transmission can be reduced by improving housing conditions, including adequate and timely housing repair and maintenance, and the enabling environment to perform healthy behaviours.

## 1. Introduction

*A healthy home is a fundamental precondition of a healthy population. Important contributors to the current unsatisfactory living conditions include inadequate water and sewerage systems, waste collection, electricity and housing infrastructure (design, stock and maintenance). Children need to live in accommodation with adequate infrastructure conducive to good hygiene and study, and free of overcrowding.*

(‘Healthy Homes’, Australian National Indigenous Reform Agreement (2009) [1])

Housing maintenance remains an enduring health policy challenge in remote Indigenous Australian communities. Unmaintained housing and malfunctioning ‘health hardware’, such as bathroom, kitchen and laundry facilities, can inhibit or prevent water-based hygiene practices [2,3,4,5,6]. Inadequate hygiene practices can in turn promote the spread of infectious disease, including intestinal, eye, ear, and skin infections; and ultimately result in chronic sequelae, such as stunting, blindness, hearing loss, rheumatic heart disease and renal failure [7]. In Indigenous Australian communities, the persistence of trachoma is emblematic of the health consequences of unresolved housing challenges: the condition is preventable through facial hygiene and environmental improvements, yet Australia remains the only developed nation in the world where trachoma is endemic [8,9]. The burden of infectious disease among Indigenous Australians is particularly evident in the Northern Territory, where shigellosis notifications have recently increased to the highest level this century, reported incidence rates for acute rheumatic fever have risen since 2006, and trachoma prevalence remains the highest in the country [10,11,12].

This research focuses on Indigenous Australians as their overall life expectancy is at least 10 years less than non-Indigenous people [13]. The focus is Australia wide, as the majority of the approximately 687,000 Indigenous Australians live in urban areas, with only 21 percent residing in remote or very remote areas [14]. Of note geographically, however, is that remote Indigenous communities experience higher rates of these infectious diseases, which is linked to crowding through insufficient housing stock in these communities [15]. Assessments of the health consequences of poor housing have focused on a infectious diseases (intestinal, skin, eye, respiratory) [16,17] where data on both hospital admissions and clinical presentations demonstrate a heavy infectious disease burden borne by Indigenous households that are also noted to be crowded [18,19,20]. This is due to crowded conditions resulting in faster break downs in health hardware, especially if this hardware is low quality and not fit for purpose [15]. The national plan for housing notes that investment in quality housing and in regular and proactive maintenance can significantly reduce infection rates [15].

These health inequalities between Indigenous and non-Indigenous Australians are the focus of the Government’s ten-year campaign to ‘close the gap’ in health, education and other inequalities. The contribution of quality and functional housing (and health hardware) are noted in the Government’s plan as supporting the health, education, employment, and community safety targets [21]. This approach recognises that housing is a social determinant of health in terms of lower socio-economic status groups tending to live in sub-standard housing, the relationship of Indigenous people to living on their traditional country (that can be remotely-located) and the ongoing policy and legacy impacts of colonisation [22].

In response, $5.5 billion has been invested in public housing in remote communities over the past 10 years through the National Partnership Agreement for Remote Indigenous Housing (NPARIH, now the Remote Housing Strategy) [15]. NPARIH sought to improve the quality of housing, reduce chronic crowding, and complete repairs within shorter timeframes and with cyclic (not reactive) maintenance, with a focus on health related hardware and house functionality [15,21].

This research seeks to identify systemic housing-level contributions to infectious disease transmission in Indigenous Australian households, in contrast to the traditional ‘vertical’ approach of medical interventions to infectious diseases [23]. The intention is to identify the contribution of social determinants and geographic disparities to infectious diseases in the Indigenous Australian population.

## 2. Materials and Methods

This study sourced data through a narrative literature review, defined as a comprehensive syntheses of previously-published material [24]. The narrative review method was selected as optimal considering the high variability in the outcome measures and methodology of the literature as well as the broad scope of the research topic and the wide range of issues it encompasses [25].

A database search of English language literature published in the last 10 years (between 1 January 2008 and 31 July 2018) was performed using three electronic databases: MEDLINE, Web of Science, and PubMed. The search strategy was performed in accordance to Preferred Reporting Items for Systematic reviews and Meta-Analyses (PRISMA) guidelines [26]. Search terms were categorized into four groups corresponding to the scope of the study: health, environment, people, and place. Health terms addressed infection and communicable diseases, as well as specific illness identified as prevalent in Indigenous Australian populations. Environment terms addressed the residential built environment, including dwelling condition, health hardware, and overcrowding. Health hardware was defined as the equipment that households need to carry out healthy living practices [27]. People and place terms focused on Aboriginal and Torres Strait Islander Peoples residing in Australia. Filters for humans were applied. A manual search of additional articles was conducted using the ‘snow-balling’ technique that investigated relevant citations in the included literature [28].

In addition, ‘grey’ literature was identified through a web search using Google Scholar, and a manual review of government websites. Despite the limited use of grey literature sources in similar reviews, its inclusion in this review was due to the resulting and limited number of formal literatures on the topic, the ability to consider outcomes of more recent or yet-to-be-published projects, and the strong role played by government departments gathering data [29].

**Figure 1:**
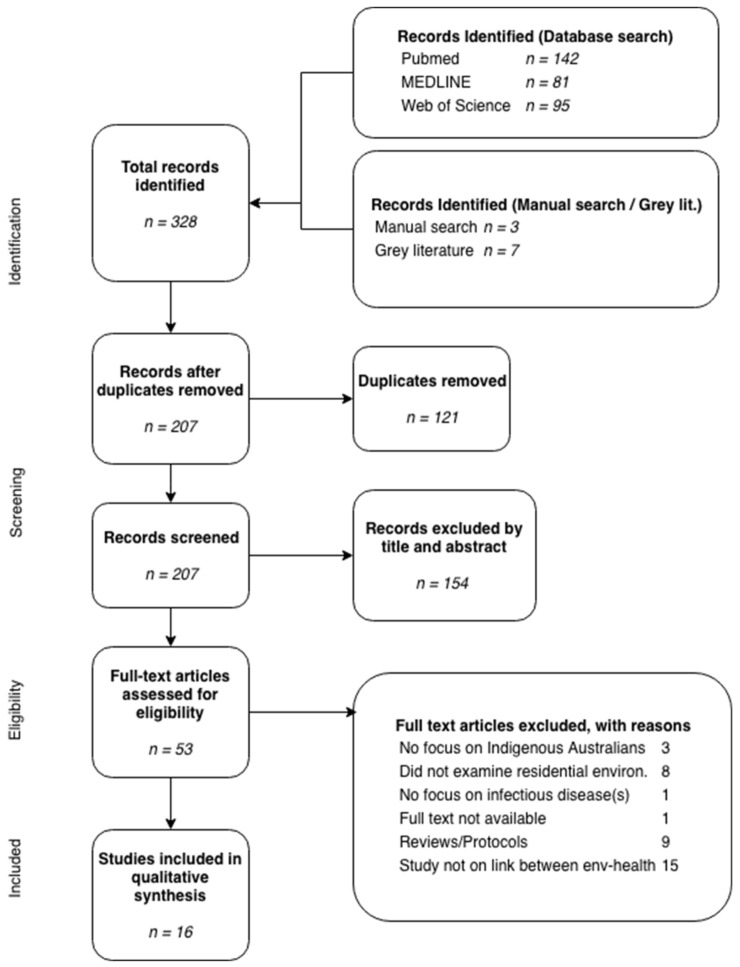
PRISMA flow-chart of search strategy and results.

After duplicates were removed, a preliminary filtration using titles and abstracts to identify articles for full text review was conducted. Articles studying the health of Indigenous Australian peoples irrespective of age or gender were within the scope of this review. A study was included for full text review under the condition that it abided by the following criteria. Studies were excluded if it was manifestly clear that the paper did not concurrently address the four thematic domains (health, environment, people and place). Included studies likewise examined infectious diseases defined by the World Health Organization (WHO) as “diseases caused by pathogenic microorganisms, such as bacteria, viruses, parasites or fungi …(that can) spread, directly or indirectly, from one person to another” [30]. Studies that were not in English, were reviews, protocols, or abstracts, did not have full-texts available, or did not explicitly examine a correlative or causative link between the residential environment and health, were excluded. The studies that passed the full-text review underwent a data-extraction process using categories established from a survey of the included literature. This process sought to identify information pertaining to the study methodology, population characteristics of the studied individuals, general study setting (i.e., urban or rural as defined by the Australian Bureau of Statistics [31]), what types of infectious diseases were studied and how they were examined, how residential environment factors were evaluated, and any broad observations made. In total, 328 articles were identified through the initial database search, three through an additional manual search (snowballing method), and 10 through a grey literature search. The 210 studies remaining after the removal of duplicates were then screened for relevance. Fifty-six articles were then selected for full-text review, and a subsequent 37 articles were excluded due to: no focus on Indigenous Australians (*n* = 3), did not examine an aspect of the residential environment (*n* = 8), did not focus on infectious diseases (*n* = 1), full text was not available (*n* = 1), study was a review, protocol, or abstract (*n* = 9), or did not explicitly examine the link between residential environment and infectious diseases (*n* = 15). The resulting 16 articles included in this review synthesis included 14 published studies and two pieces of grey literature. These results are provided in Figure 1 in a flow diagram format that follows the reporting guidelines of PRISMA, the internationally-agreed visual presentation that reflects the flow of information through the different phases of a review [26].

## 3. Results

### 3.1. Study Characteristics

The resulting 16 publications analysed for this study varied significantly in design, and included five cohort studies [32,33,34,35,36], three cross-sectional studies [16,37,38], three qualitative descriptive studies [39,40,41], two case studies [42,43], government-commissioned research summary and cohort study [44,45], and one modelling study [46]. Geographically, 10 studies (62.5%) focused on rural communities [33,35,37,39,40,41,42,43,46]. The Northern Territory was the most studied region (*n* = 6) [16,34,39,41,43,46], followed by Western Australia (*n* = 4) [32,33,35,37], New South Wales (*n* = 2) [36,44], Multi-state (*n* = 2) [37,45], Queensland (*n* = 1) [38], and South Australia (*n* = 1) [42]. These are described in Table 1.

### 3.2. Housing Environment

Various aspects of the residential environment were surveyed. However, many studies (43.8%, *n* = 7) relied upon self-reported surveys to assess housing conditions. One study tested an innovative visual-based method of data collection. Of the 10 studies examining functionality, six studies actively assessed house function (through infrastructure surveys conducted by trained individuals) based on infrastructure required for the nine Healthy Living Practices (HLPs), which include (1) washing people; (2) washing clothes and bedding; (3) removing waste water safely; (4) improving nutrition and the ability to store, prepare, and cook food; (5) reducing the negative impacts of over-crowding; (6) reducing the negative effects of animals, insects; and vermin, (7) reducing the health impacts of dust; (8) controlling the temperature of the living environment; and (9) reducing hazards that cause trauma [3].

Four studies examined aspects of health hardware (including functionality of showers, toilets, and electrical systems, taps, and stoves), infestation or presence of animals in the house, temperature, and external connections to sewage systems. Crowding data was a focus of the vast majority of the included studies (87.5%, *n* = 14), and was commonly obtained through survey questions on the number of people sleeping in the home; the number of people per bedroom; whether other children (besides study subjects) lived in the home; whether eight or more people lived in the house; whether there was more than one person per room; or whether the study subjects felt crowded in the past 12 months.

Among the studies assessing crowding levels, the number of people per household study ranged from six [37] to eleven [34]. In two studies [33,41], the majority of households studied were classified as crowded, defined as more than an average of 1 person per room [33] or more than two persons per each available bedroom [41]. However, one case study noted that numeric crowding indices did not provide a full picture of the nature of crowding and its impact on health: once mobile, children often choose to sleep with certain family members regardless of the number of available bedrooms [41].

Housing condition and health hardware functionality remained a key concern. One study noted that 89.3% of surveyed households had one or more items of HLP-related infrastructure requiring major or urgent repair, and 42.2% of households with children had faeces or other decaying matter in the immediate living environment [39]. Another study identified crowding and inadequate infrastructure as major concerns amongst the interviewed Indigenous Australian community [40]. Fifty-nine percent of communities in another study rated their housing unsatisfactory or very unsatisfactory, with issues pertaining to dust, water, and sanitation/waste being identified as key environmental concerns [37]. One article found nearly 40% of respondents indicating housing problems or the need for home repairs [45].

In longitudinal studies, the overall improvement in household infrastructure and functionality was somewhat limited. In one study, 25% of children witnessed an improvement of two or more points in the Surveyor Function Score (SFS) and 38% of children witnessed the same improvement in the number of failed HLPs [34]. In another study involving an environmental intervention which included (among other activities) replacing poorly built houses, biweekly trash collection, upgrades to sewage and water lines, and installation of rainwater tanks, improvement was only witnessed in 3 of the 8 examined HLPs [42]. Nonetheless, the New South Wales (NSW) Housing for Health program outlined in one study led to improved household function across all examined environmental indictors [44]. Household residents who were provided with health hardware repairs under the program were compared for hygiene-related infectious disease conditions—notably respiratory infection, skin infection, intestinal infection and otitis media [15]. It was found that the program recipients had a rate of hospital separations for these diseases that was 40 percent lower than non-recipients [15].

### 3.3. Link between Housing and Infectious Disease

The primary categories of infectious diseases studied included gastrointestinal, skin, ear, eye, and respiratory illnesses. Here, the presence of an ‘association’ is taken as given based on conclusions of the various studies. Residential environmental factors found to be positively associated with infectious diseases in Indigenous Australian populations are summarised in Table 2. Ear-related illnesses were the most common condition assessed in the included studies [16,32,33,34,37,38,45,47], followed by skin-related illnesses [16,34,35,37,45,47] and gastrointestinal illnesses [34,35,36,37,47].

The link between crowding variables and gastrointestinal infection varied across studies; one study found crowding to be strongly associated with gastrointestinal infection (Crude Odds Ratio (cOR) 3.51; *p =* 0.004) [37], while another found no statistically significant association (although a link between carer reports of feeling crowded were found to be associated with gastrointestinal infection, Adjusted Prevalence Ratio (aPR) 1.63; *p =* 0.05) [36]. Counterintuitively, having six or more people sleeping in a home was protective against recurrent gastrointestinal infection (aPR 0.56, *p =* 0.05) [36]. Major structural problems (aPR 2.42; *p =* 0.05), major plumbing problems (aPR 1.95; *p =* 0.05), and damp or mildew (aPR 1.82; *p =* 0.05) showed positive associations with gastrointestinal infection [36]. Infectious skin disease was found to be associated with crowding (cOR 2.71 (reported crowding as environmental risk factor), *p =* 0.007 [37]; cOR 2.92 (3–5 adults per room vs. <3 adults per room), *p =* 0.05 [16]; cOR 3.96 (7–8 adults per room vs. <3 adults per room), *p =* 0.05 [16]; (Adjusted Odds Ratio) aOR 1.81 (reduction of 2 or more persons per bedroom), *p =* 0.28 [34]). Skin infections were positively associated with the greatest number of environment indicators across the six disease categories. The link between ear infections and residential environment factors was also well established, including with crowding variables (OR c3.01 (reported crowding as environmental risk factor), *p* = 0.001 [37]; OR 2.67 (>1 person per room), *p =* 0.021 [33]; aOR 3.8 (8 or more people in household), *p =* 0.05 [38]; aOR 1.45 (each child in addition to the study participant in a 4-room house), *p =* 0.05 [32]) as well as poor toilet infrastructure (cOR 2.26, *p =* 0.05) [16] and conditions of bedding and sleeping areas (cOR 4.81, *p* < 0.05) [16]. Eye infections were the least studied across the six disease categories and the evidence linking them with housing was inconclusive; while one study identified a significant association between eye infections and crowding (cOR 3.01 (reported crowding as environmental risk factor), *p =* 0.001) [37], a prospective observational study examining the impact of environmental improvements on trachoma found no significant association [42]. Among the other disease-environment links studied, diarrhoea/vomiting was shown to be associated with the hygienic state of food preparation and storage areas (cOR 2.10, *p =* 0.05) [16], and viral conditions such as influenza were associated with crowding (cOR 3.31 (reported crowding as environmental risk factor), *p* < 0.001) [37].

Despite the various specific environmental factors linked with infectious diseases, the overall condition of a house remained an important determinant of infectious disease rates; one study noted that the number of housing problems mattered more than any specific issue, since each additional issue may present a greater risk for pathogen exposure [36]. Moreover, while a number of associations between the residential environment and health were made across the studies, the specific causal pathways for some of these associations still remained unclear; one study noted that the correlation identified between mildew, temperature control, and major structural problems and gastrointestinal infection had limited background evidence to support a potential causative link [36].

Factors outside the residential environment played pivotal roles in influencing the health status of the studied populations and potentially the link between housing and health. Socio-economic factors [16,34,39], food insecurity [35], and the psychosocial status of carers in the context of child health [16] were shown to impact many of the study populations examined in the included literature. Barriers of rural environments (including limited resource connectivity) [35] and the degree to which children in the community or neighborhood interacted with each other [34] likewise influenced infectious disease transmission rates. The authors of one study noted that increased population size of a community (such as those in urban environments) was associated with more reported environmental risk factors and health issues, which they attributed greater land engagement and ‘caring of the country’ in smaller communities [37]. Moreover, environmental interventions seeking to translate into real improvements in health must consider whether the intervention will be well received by community members [41], the sustainability of government commitment [39], and the initial specifications of health hardware [43].

## 4. Discussion

The results revealed that that the housing environment and infectious diseases exist in a complex and interlinked system where social determinants (reflected in housing options and quality) and geographic disparities (reflected in chosen locations of homes on traditional country) contribute significantly to health outcomes. Housing factors and crowding may interact with each other and can be exacerbated by broader socio-economic and cultural factors acting upon the residential environment. This systems thinking reflects an understanding of the relationship between system structure and the dynamic behaviour of a system [48,49]. A systems thinking approach is based on the central concept of understanding how reinforcing (positive) and balancing (negative) feedbacks can combine to link structure to the dynamic behaviour of an integrated system [50]. It is introduced here to assist understanding of the relationship between health and housing as revealed by the reviewed studies.

As examples of a dynamic system that links both housing functionality and infectious disease transmission, housing and crowding were two factors shown to have links. One study noted that structural issues often exacerbated the impact crowding had on health by limiting residents ability to perform daily tasks in a crowded home [36], while another noted that issues of crowding also limited the success of building programs seeking to improve the housing [34]. The role of hygiene and protective behaviors such as handwashing within the dynamic of housing and health was of pivotal importance. The link between hygiene practices and health has been well-studied [11]. Indeed, the impact of reduced crowding or infrastructure improvements on improving health was found to be limited by domestic hygiene practices [34,43]. However, good hygiene was shown to generally be already valued [43], and environmental factors acted as key enabling tools for improved hygiene [34,39,41]. Likewise, health awareness and having the necessary skills and knowledge of maintaining appropriate hygiene and ensuring the sustainably of the health-enabling environment was significant [41].

**Figure 2:**
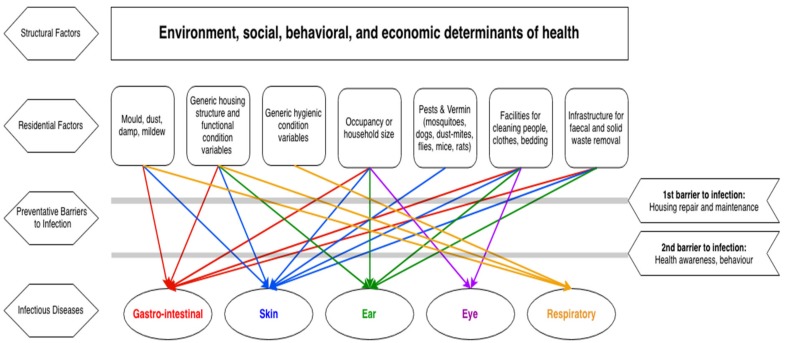
Systems approach to explain links between housing and infectious diseases. Sources: [32,33,34,35,36,37,38,46].

These results regarding the systemic nature of the relationship between housing and infectious disease are visually displayed in Figure 2. These results are based both on the empirical results of the included studies and the wider analysis presented in this section. At the top level are structural aspects that contribute to health, including social, behavioral, and economic factors. In turn, these contribute to specific residential factors, as identified in the literature. This includes the presence of functioning health hardware, infrastructure for sewage removal and management, household population size, pests and vermin, and mould and mildew. Each of the residential factors can create the conditions for infectious disease transmission. For example, gastrointestinal infections are associated with the presence of mould and mildew, crowding, lack of infrastructure to wash people, clothes and bedding, and safe removal of faecal matter. Skin infections are associated with all of the identified residential factors. Ear infections are associated with crowding, lack of functioning facilities for washing people and bedding and sewage removal. Eye infections are associated with crowding. Respiratory infections are associated with mould and mildew, and more generic housing and hygiene factors. However, these residential factors can be prevented from exacerbating or contributing to infection through housing repair and maintenance. In turn, functioning health hardware enable the secondary barrier of healthy behaviours.

There were several limitations in this study. Firstly, the minimal amount of research which fell within the scope of the study limited the ability to make strong conclusions about the link of housing and infectious diseases in Indigenous Australian sub-populations. Secondly, a systematic quality assessment of the included literature was unable to be conducted given the significant variation in study methodologies, although the methodological rigor and overall quality of the included studies was also limited. Finally, multivariable results were generally more robust than univariable. Where a significant univariable association was found, the association did not always remain significant when adjusted for other covariates. This limited the value of the univariate studies.

## 5. Conclusions

In response to the opportunities to ‘close the gap’, this research analysed publications examining the contribution of housing and infectious disease transmission for Indigenous Australians in urban, rural and remote settings. These two aspects are closely linked to the social determinants of health and the geographic location of homes- especially those in remote communities. The primary categories of infectious diseases included gastrointestinal, skin, ear, eye, and respiratory illnesses. Poorly maintained housing and the state of food preparation and storage areas were associated with gastrointestinal infections. Skin-related diseases and viral conditions such as influenza were associated with crowding. Gastrointestinal, skin, ear, eye, and respiratory illnesses, were all related in various ways to functional health hardware, removal and treatment of sewage, crowding, presence of pests and vermin, and the growth of mould and mildew. These relationships can be expressed as a system, where social, behavioral, and economic determinants of health contribute to specific residential factors. The evidence suggests the burden of infectious disease among Indigenous Australians can be reduced through improved housing conditions, adequate and timely housing repair and maintenance, and the ability to perform healthy behaviours. These findings reiterate the importance of a proactive approach to prevent disease transmission rather than relying on later medical interventions to contribute to closing the health gap between Indigenous and non-Indigenous Australians.

## Figures and Tables

**Table 1:** Characteristics of studies meeting inclusion criteria.

Reference	State/Territory	Study Design	Area (Rural/Urban)
Andersen et al., (2018) [36]	NSW	Cohort Study	Urban
Bailie et al., (2012) [34]	NT	Cohort Study	Mixed
Bailie et al., (2010) [16]	NT	Cross-sectional Study	Mixed
Dossetor et al., (2017) [35]	WA	Cohort Study	Rural
Jacoby et al., (2008) [33]	WA	Cohort Study	Rural
Lansingh (2010) [42]	SA	Case Study	Rural
Massey et al., (2011) [40]	Multiple	Qualitative Descriptive Study	Rural
McDonald, E.; Bailie, R. [39]	NT	Qualitative Descriptive Study	Rural
McDonald et al., (2010) [41]	NT	Qualitative Descriptive Study	Rural
McDonald (2009) [43]	NT	Case Study	Rural
Melody et al., (2016) [37]	WA	Cross-sectional Study	Rural
Spurling et al., (2014) [38]	QLD	Cross-sectional Study	Urban
Vino et al., (2017) [46]	NT	Modelling Study	Mixed
Jacoby et al., (2011) [32]	WA	Cohort Study	Rural
Sartbayeva (2016) [45]	Multiple	Research summary	Mixed
NSW Department of Health (2010) [44]	NSW	Cohort study	Rural

* NSW: New South Wales; NT: Northern Territory; WA: Western Australia; SA: South Australia; QLD: Queensland.

**Table 2:** Summary of link between infectious diseases and residential environment.

Disease Categories	Reported Association with Residential Environment
Gastrointestinal (e.g., intestinal infections)	**POSITIVE ASSOCIATION:** Crowding [37], Perception of crowding [36], structural or plumbing problems [36], damp or mildew [36], houses in need of repair/improvement [44], low availability of household cleaning equipment [34], diarrhoea/vomiting associated with poor hygienic state of food preparation and storage areas [16] **NO/MINIMAL ASSOCIATION:** Crowding [36], water quality [37]
Skin (e.g., skin infections, scabies)	**POSITIVE ASSOCIATION:** Crowding [16,34,37], poor temperature control [16], evidence of pests and vermin [16], houses in need of repair/improvement [45,47], infrastructure required to wash clothes and bedding [34] *, prepare and store food [34], remove human waste [34], remove rubbish [16,34], control mold [34], control dust [16] **NO/MINIMAL ASSOCIATION:** [for skin infection] Any infrastructure variables [16], water quality [37]
Ear (e.g., ear infections, hearing)	**POSITIVE ASSOCIATION:** Crowding [32,33,37,38], limited toilet infrastructure [16], houses in need of repair/improvement [16,47], poor hygienic condition of bedding and sleeping areas [34]
Eye (e.g., trachoma)	**POSITIVE ASSOCIATION:** Crowding [37] **NO/MINIMAL ASSOCIATION:** Environmental improvement [42]
Respiratory (e.g., lung infections)	**POSITIVE ASSOCIATION:** Poor overall hygienic condition of house [16], poor overall function of house [16], houses in need of repair/improvement [44], dust [37], crowding/crowding associated with influenza, flu/cold transmission [37,40,46] **NEGATIVE ASSOCIATION:** Infrastructure required for ‘wash people’ variable [37]

* Note: improvement in clothes washing do not necessarily drive health improvements if crowding remains high.
